# DMM community consultation: help us plan for the next 10 years

**DOI:** 10.1242/dmm.037507

**Published:** 2018-10-24

**Authors:** Rachel Hackett, O. Claire Moulton

**Affiliations:** Disease Models & Mechanisms, The Company of Biologists, Bidder Building, Station Road, Histon, Cambridge CB24 9LF, UK

Ten years ago, the directors of The Company of Biologists, all practising scientists, made the decision to launch a new journal aimed at researchers who investigate human disease using model organisms (Freeman and St Johnston, 2008). They believed that the application of fundamental discoveries from model organisms would accelerate our understanding, diagnosis and treatment of human diseases. The directors and newly appointed editorial team, led by Vivian Siegel (Siegel, 2008), hoped that this journal, named Disease Models & Mechanisms (DMM), might help define an important new field and provide a service to the scientists moving into it.

**Figure:**
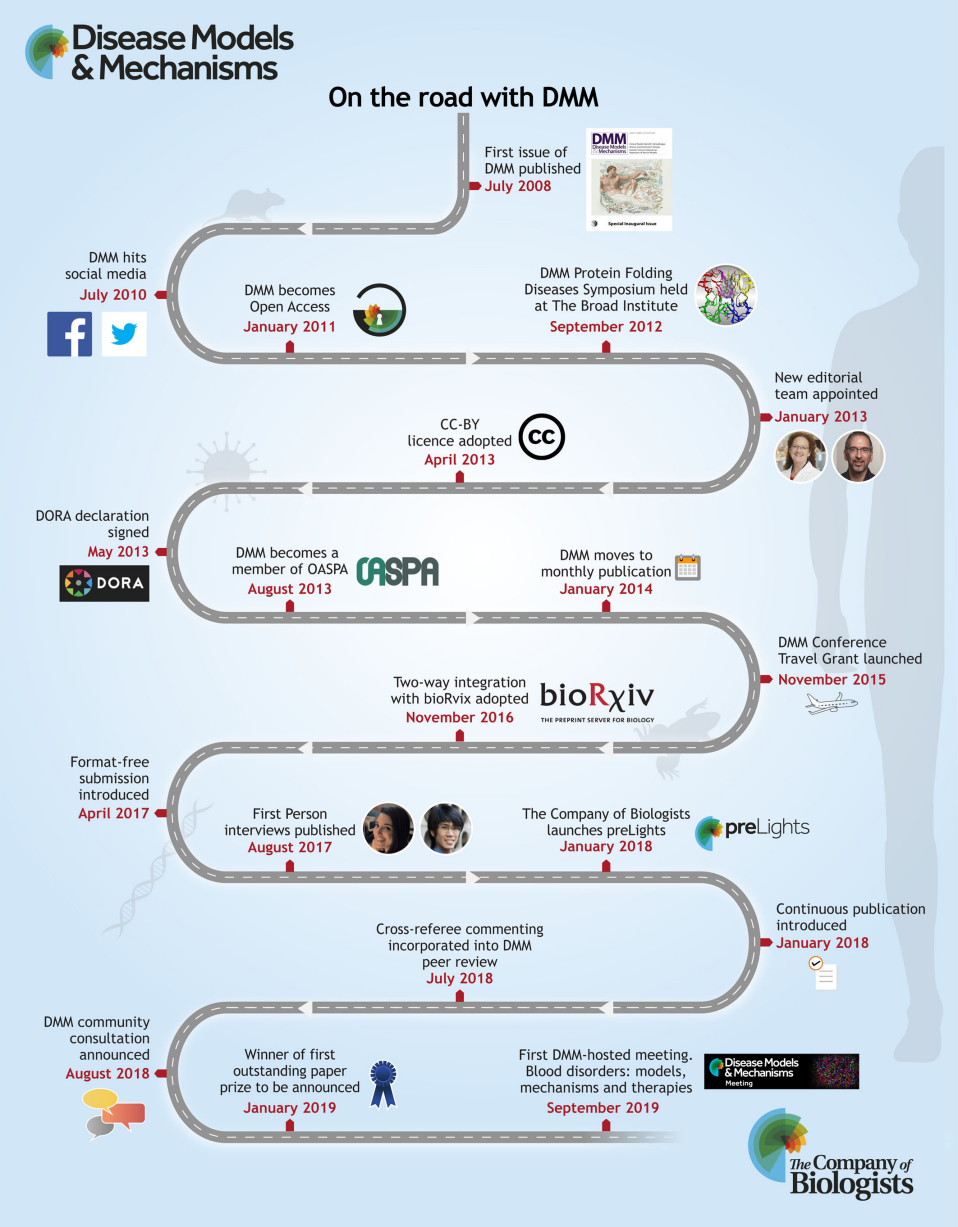

Biomedical research journals such as DMM sit in an ecosystem that is subject to alterations from various sources – technological, financial and policy, in addition to advances in the science itself. The academic editors of DMM, currently led by Editor-in-Chief Monica Justice and Senior Editor Ross Cagan, work closely with the editorial team in Cambridge, UK, to ensure that DMM serves its community of readers, authors and reviewers as effectively as possible within this ecosystem (Cagan et al., 2013).

As the journal marked its first decade, it was agreed that the long-term editorial succession and journal strategy should be reviewed. Because DMM strives to be a journal that serves its community, it was therefore obvious that any decision about the future of DMM must involve discussion with its authors, reviewers and readers. A plan was initiated for a community consultation regarding the future direction of DMM.

This consultation will be steered by a subgroup of Company directors: Peter Rigby, Daniel St Johnston and Paresh Vyas. As you will see, the consultation includes gathering suggestions for a future Editor-in-Chief. This should be someone who will maintain DMM's high standards for model organism work, while promoting new fields, to ensure that the journal is forward-looking and reflects the directions of the DMM field. It should be someone who has a medical disease focus, and whose work bridges the gap between basic research and clinical science (from bench to bedside). They might perhaps be someone who has shown commitment to DMM (as author, referee, editor, etc.) over a period of time.

As well as the key matter of the Editor-in-Chief role, we also want to consult the community about the strengths and weaknesses of DMM, where we can improve, which (new or existing) areas the journal should expand into and what more we can do to support our field (see Box 1). We welcome all your thoughts on these aspects in particular, although free-form feedback will also be considered! Please contact us via dmm.feedback@biologists.com or get in touch with any of us individually.

**Box 1. DMM community consultation: have your say**

What are the strengths of DMM?

What weaknesses does DMM have?

What niche does DMM currently occupy?

Into which (new or existing) areas would you like to see DMM expand in the future?

Is DMM seen as a community journal?

What else could DMM do to support our field? (See http://dmm.biologists.org/content/about#COMMUNITY.)

Your suggestions for new Editor-in-Chief (with brief reasons please).

Although a mere stripling compared with our more venerable sister journals (Development, Journal of Cell Science and Journal of Experimental Biology), the ten years since journal launch have seen substantial change. DMM was launched as a print and online subscription journal, at a challenging time for library budgets and when the campaign for improved access to research was flourishing. The directors of The Company of Biologists made the decision to move to an author-pays Open Access model and to support DMM through what could have been a difficult transition (Siegel, 2011). Happily, DMM has thrived, with more submissions leading to more published articles and necessitating the move to monthly publication following its original bimonthly release. As a not-for-profit publisher that uses the surplus it generates to benefit the biological community, the directors launched DMM not for commercial advantage, but because it was the right thing to do. This commitment and ethos remains unchanged.

As well as the day-to-day decisions made concerning submissions to the journal, the team thinks more strategically; for example, deciding which areas to devote to special issues of DMM and evaluating the current scope of the journal to ensure that it keeps pace with the science. They make decisions about the operation of the journal, such as the recent move from issue-based article publication to continuous publication, and offering format-free submission. In an Editorial published in February 2018 (Hackett, 2018), we promised that DMM would be looking at the peer review process, and we have, adopting a more collaborative form of peer review. DMM peer reviewers now have a 48-h window during which they are invited to comment on the other referee reports before the editor makes a decision. The aim of this ‘cross-referee commenting’ step is to help resolve differences between referees, identify unnecessary or unreasonable requests, or – conversely – highlight valid concerns raised by one referee but overlooked by others. Launched in July, we have seen comments that are enormously useful to our editors in ensuring constructive and fair peer review. DMM is also closely following arguments in support, or otherwise, of publishing peer review reports. We welcome additional feedback on our publishing policies.

DMM has kept an eye on discussions about the role of preprints in science communication and has long had preprint-friendly policies, including the two-way integration of its submission system with bioRxiv. DMM readers (as well as the entire biological sciences community, we hope) have been beneficiaries of the launch of preLights – the new preprint highlights service run by the biological community and supported by The Company of Biologists. A team of scientists, several of whom were recommended by DMM Editors and Editorial Board members, regularly review, highlight and comment on preprints they feel are of interest to DMM. You will now find preprint highlights relevant to DMM on our tables of contents; do let us know if you are finding this useful. We await the launch of MedArXiv with curiosity, especially given the debate within the medical community about possible risks arising from posting non-peer-reviewed clinical research.

DMM is extremely lucky to have both Monica Justice and Ross Cagan as its senior leadership; both will remain in place while we gather and review the feedback over the coming months. It is vastly more important to do this right than do it quickly, to ensure that DMM continues to publish research that enhances our understanding of disease mechanisms and the development of novel diagnostics and therapeutics.
